# The Dynamics of Neural Fields on Bounded Domains: An Interface Approach for Dirichlet Boundary Conditions

**DOI:** 10.1186/s13408-017-0054-4

**Published:** 2017-10-26

**Authors:** Aytül Gökçe, Daniele Avitabile, Stephen Coombes

**Affiliations:** School of Mathematical Sciences, University of Nottingham, University Park, Nottingham, NG7 2RD UK

**Keywords:** Neural fields, Bounded domain, Dirichlet boundary condition, Interface dynamics, Piece-wise constant kernel

## Abstract

Continuum neural field equations model the large-scale spatio-temporal dynamics of interacting neurons on a cortical surface. They have been extensively studied, both analytically and numerically, on bounded as well as unbounded domains. Neural field models do not require the specification of boundary conditions. Relatively little attention has been paid to the imposition of neural activity on the boundary, or to its role in inducing patterned states. Here we redress this imbalance by studying neural field models of Amari type (posed on one- and two-dimensional bounded domains) with Dirichlet boundary conditions. The Amari model has a Heaviside nonlinearity that allows for a description of localised solutions of the neural field with an interface dynamics. We show how to generalise this reduced but exact description by deriving a normal velocity rule for an interface that encapsulates boundary effects. The linear stability analysis of localised states in the interface dynamics is used to understand how spatially extended patterns may develop in the absence and presence of boundary conditions. Theoretical results for pattern formation are shown to be in excellent agreement with simulations of the full neural field model. Furthermore, a numerical scheme for the interface dynamics is introduced and used to probe the way in which a Dirichlet boundary condition can limit the growth of labyrinthine structures.

## Introduction

Neural field models are now widely recognised as a natural starting point for modelling the dynamics of cortical tissue. Since their initial inception in the 1970s by Wilson and Cowan [1, 2], Amari [3, 4], and Nunez [5], they have been extensively studied in idealised one-dimensional or planar settings, which are typically either infinite or isomorphic to a torus. This has facilitated both the mathematical and the numerical analyses of spatio-temporal patterns, and much has been learnt about localised states, global periodic patterns, and travelling waves. Indeed there are now a number of reviews summarising work to date, such as [6–9], and how neural field modelling has shed light on large-scale brain rhythms, geometric visual hallucinations, mechanisms for short term memory, motion perception, binocular rivalry, and anaesthesia, to list just a few of the more common application areas. For the most recent perspective on the development and use of neural field modelling we recommend the book by Coombes *et al*. [10], which also includes a tutorial review on the relevant mathematical methodologies (primarily drawn from functional analysis, Turing instability theory, applied nonlinear dynamics, perturbation theory, and scientific computation). This substantial body of knowledge is still expanding with further refinements of the original neural field models to include other important aspects of cortical neurobiology, including axonal delay [11], synaptic plasticity [12], and cortical folding [13], as well as rigorous mathematical results for existence and uniqueness of stationary solutions on bounded subsets of $\mathbb{R}^{n}$ without regard to imposition of boundary conditions [14], and new numerical algorithms for their evolution and numerical bifurcation analysis [15, 16]. Neural field models are typically expressed in the form of integro-differential equations, whose associated Cauchy problems do not require the specification of boundary conditions. The value attained by the activity variable at the boundary is determined by the initial condition and by the non-local synaptic input. However, very little work has been done on the enforcement of boundary conditions in neural fields, or on their effect on inducing patterned states. An exception to this statement is the work of Laing and Troy [17], who proposed an equivalent partial differential equation (PDE) formulation of the neural field equation. While boundary conditions must be specified in the PDE setting, they are often chosen to ensure the smooth decay of localised solutions rather than model any biophysical constraint. It is already appreciated that the continuum neural fields can be extended to include different properties that can strongly influence the spatio-temporal dynamics of waves and patterns. For example, heterogeneities may give rise to wave scattering [18] or even extinction [19]. The topic we address in this paper is to ponder the role that a boundary can have on spatio-temporal patterning. Given the historical success of analysing neural fields with a Heaviside firing rate, our first step in this direction will be taken within the so-called “Heaviside world” of Amari [20]. Amari’s seminal work developed an approach for analysing localised solutions of neural field models posed on the real line, and has recently been extended to the planar case by Coombes *et al*. [21], albeit assuming that the synaptic connectivity can be expressed in terms of a linear combination of zeroth order modified Bessel functions of the second kind. This approach is not only able to describe localised stationary solutions, often called bumps in one dimension and spots in two dimensions, but also dynamically evolve states such as travelling pulses and their transients as well as spreading labyrinthine patterns. Since the Amari approach, in either one or two spatial dimensions, effectively tracks the boundary between a high and low state of neural activity, where the firing rate switches, we shall refer to it as an *interface dynamics*. Importantly it gives a reduced description of solutions to a neural field model without any approximation. On the down side the approach cannot be generalised to treat smooth firing rate functions, though simulations by many authors have shown that the behaviour of the Amari model is consistent with neural field models utilising a steep sigmoidal function. Here we show how the interface dynamics approach can be generalised to include the effects of Dirichlet boundary conditions for arbitrary choices of synaptic connectivity.

In Sect. 2 we introduce a simple scalar neural field model in the form of an integro-differential equation defined on a finite domain, and discuss natural boundary conditions for neural tissue. Focussing on Dirichlet boundary conditions we develop the key mathematical idea in this paper. Namely that the re-formulation of the original scalar model in terms of the evolution of its gradient allows for an interface description that respects Dirichlet boundary conditions. To illustrate the effectiveness of this approach we first treat the example of localised states in a one-dimensional model in Sect. 3. This is a useful primer for the construction of an interface dynamics in a two-dimensional model, presented in Sect. 4. The first part of Sect. 4 also shows how to generalise the original treatment in [21], for infinite domains, to handle arbitrary choices of the synaptic connectivity function (removing the restriction to combinations of Bessel functions). Localised bump and spot solutions of the interface dynamics are explicitly constructed and their stability determined. In Sect. 5 we extend this approach to treat Dirichlet boundary conditions, and in Sect. 6 we show explicitly how this approach can be used to handle spots and their azimuthal instabilities. We work with standard Mexican hat synaptic connectivities, as well as piece-wise constant caricatures for which calculations simplify. All our theoretical results are found to be in excellent agreement with direct simulations of the original neural field model. We also develop a numerical scheme to evolve the interface dynamics and use this to highlight how a Dirichlet boundary condition can limit the growth of a spreading pattern arising from the azimuthal instability of a spot. Finally in Sect. 7 we discuss natural extensions of the work in this paper.

## A Neural Field Model with a Boundary Condition

Although single neuron models are able to predict dynamical activity of real neurons that have a wide variety of spiking behaviour [22, 23], they are not well suited to describe the behaviour of tissue on the meso- or macro-cortical scale. To a first approximation the cortex is often viewed as being built from a dense reciprocally interconnected network of cortico-cortical axonal pathways [24]. These fibres make connections within the roughly 3 mm outer layer of the cerebrum. Given the large surface area of the (folded) cortex (∼800–$1500~\mbox{cm}^{2}$) and its small depth it is sensible to view it as a two-dimensional surface. Neural field modelling, on a line or a surface, is a very well-known framework for capturing the dynamics of cortex at this coarse level of description [10]. As well as being relevant to large-scale electroencephalogram (EEG) and magnetoencephalogram (MEG) neuroimaging studies [8], the understanding of epileptic seizures [25], visual hallucinations [26, 27], and neural spiral waves [28, 29], they have also been used to investigate localised states linked to short term working memory in the prefrontal cortex [30, 31]. In the latter regard the idealised neural field model of Amari has proven especially advantageous [32]. This was originally posed on an infinite domain, without regard to the role of boundary conditions in shaping or creating patterns. However, the neural circuits of the neocortex are adapted to many different tasks, giving rise to functionally distinct areas such as the prefrontal cortex (for problem solving), motor association cortex (for coordination of complex movement), the primary sensory cortices (for vision, hearing, somatic sensation), Wernicke’s area (language comprehension), Broca’s Area (speech production and articulation), etc. Thus it would seem reasonable to parcellate their functional activity by the use of appropriate boundaries and boundary conditions. Previous work by Daunizeau *et al*. [33] on dynamic causal modelling for evoked responses using neural field equations has used Dirichlet boundary conditions. Here we extend the standard Amari model with the inclusion of a finite domain with an imposed Dirichlet boundary condition that *clamps* neural activity at the boundary to a specific value. Of course other choices are possible, though this one is a natural way to enforce a functional separation between cortical areas.

The scalar neural field model that we consider is given by

1 $$ \frac {\partial u({\boldsymbol {x}},t)}{\partial t} = -u({\boldsymbol {x}},t)+ \int_{\varOmega} \,\mathrm{d} {\boldsymbol {y}} w \bigl( \vert {\boldsymbol {x}} - { \boldsymbol {y}} \vert \bigr)H \bigl[u({\boldsymbol {y}}, t)-\kappa \bigr], $$

where *Ω* is a planar domain $\varOmega\subseteq \mathbb{R}^{2}$, with $\boldsymbol {x} \in\varOmega$ and $t \in \mathbb{R}^{+}$. The variable *u* represents synaptic activity and the kernel *w* represents anatomical connectivity. For simplicity we shall only consider the case that this depends on Euclidean distance. The nonlinear function *H* represents the firing rate of the tissue and will be taken to be a Heaviside so that the parameter *κ* is interpreted as a firing threshold. We assume that a suitable initial condition is specified for (1), and we aim to impose on the corresponding solution $u(\boldsymbol {x},t)$ the Dirichlet boundary condition

2 $$ u(\boldsymbol {x},t) \vert_{\boldsymbol {x} \in\partial\varOmega} = u_{\text{BC}}, $$

where $u_{\text{BC}}$ is the prescribed boundary activity. For simplicity, we treat the case of homogeneous boundary conditions.

It was the essential insight of Amari that the Heaviside choice allows the explicit construction of localised states (stationary bumps and travelling pulses) on infinite domains, as well as the construction of these on finite domains without a boundary condition. Our key observation that allows the extension of the Amari approach to handle the boundary condition (2) is to *expose* this constraint by writing the state of the system in terms of a line integral:

3 $$ u(\boldsymbol {x},t) = u_{\text{BC}} + \int_{\varGamma(\boldsymbol {x})} \boldsymbol {z}(\boldsymbol {y},t) \cdot\,\mathrm{d}\boldsymbol {y}. $$

Here $\varGamma(\boldsymbol {x})$ denotes an arbitrary path that connects a point on the boundary to the point ***x*** within its interior, and $\boldsymbol {z} = \nabla_{\boldsymbol {x}} u \in \mathbb{R}^{2}$. An evolution equation for ***z*** is easily constructed by differentiation of (1) to give

4 $$ \frac {\partial \boldsymbol {z}(\boldsymbol {x},t)}{\partial t} = -\boldsymbol {z}(\boldsymbol {x},t) + \int_{\varOmega}\,\mathrm{d} \boldsymbol {y} \nabla_{\boldsymbol {x}} w \bigl( \vert \boldsymbol {x}-\boldsymbol {y} \vert \bigr) H \bigl[u(\boldsymbol {y},t)-\kappa \bigr], $$

with *u* given by (3).

We shall now consider Eqs. (3) and (4) as the neural field model of choice, and in the next sections develop the extension of the Amari interface dynamics. To set the scene we first consider a one-dimensional spatial model with a focus on stationary bump solutions.

## One Spatial Dimension: A Primer

Prior to describing the analysis for a two-dimensional Amari neural field model with a Dirichlet boundary condition, we first consider the more tractable one-dimensional case. This illustrates the main components of our mathematical approach, as well as delivers new results about stable boundary induced bumps.

The one-dimensional version of (3) and (4) on the finite domain $[-L,L]$ with an imposed boundary condition takes the explicit form

5 $$ z_{t}(x,t) = -z(x,t) + \int_{-L}^{L} \,\mathrm{d} y w_{x} \bigl( \vert x-y \vert \bigr) H \bigl[u(y,t)-\kappa \bigr], $$

with

6 $$ u(x,t)= u_{\text{BC}} + \int_{-L}^{x} \,\mathrm{d} y z(y,t). $$

Here $x \in[-L,L]$, $t \in \mathbb{R}^{+}$, and $u_{\text{BC}}$ denotes a constant boundary value imposed on the left end of the interval, namely $u(-L)=u_{\text{BC}}$. In passing, we note that $u(L)$ is determined once $u(-L)$ is fixed, and some choices of $u_{\text{BC}}$ will result in an even bump $u(x)$, for which $u(L) = u(-L) = u_{\text{BC}}$. We now focus on a bump solution for which $R(u) = \{ u(x) >\kappa\}$ is a finite, connected open interval. The *edges* of the bump $x_{i}(t)$, $i=1,2$, are defined by a level-set condition that takes the form

7 $$ u \bigl(x_{i}(t),t \bigr) =\kappa,\quad i=1,2. $$

We shall refer to the two bump edges as the *interface*, as they naturally separate regions of high and low firing activity. The differentiation of the level-set condition (7) generates a rule for the evolution of the interface according to

8 $$ \dot{x}_{i}(t) = -\frac{1}{z(x_{i}(t),t)} \int_{-L}^{x_{i}(t)} \partial _{t} z(y,t) \, \mathrm{d} y ,\quad i=1,2. $$

Using the second fundamental theorem of calculus we obtain an expression for the interfacial velocities

9 $$ \dot{x}_{i}(t) =\frac{(\kappa-u_{\text{BC}}) - \psi(x_{i}(t),t)+ \psi(-L,t)}{z(x_{i}(t),t)},\quad i=1,2, $$

where

10 $$ \psi(x,t) = \int_{-L}^{L} \,\mathrm{d} y w \bigl( \vert x-y \vert \bigr)H \bigl[u(y,t)-\kappa \bigr] = \int _{x_{1}(t)}^{x_{2}(t)}\,\mathrm{d} y w \bigl( \vert x-y \vert \bigr). $$

A closed form expression for $z(x,t)$ may also be found by integrating (5) using the method of variation of parameters to give

11 $$ z(x,t) = \eta(t) z_{0}(x) + \int_{0}^{t} \,\mathrm{d} s \eta(t-s) \bigl[w \bigl( \bigl\vert x_{1}(s)-x \bigr\vert \bigr)-w \bigl( \bigl\vert x_{2}(s)-x \bigr\vert \bigr) \bigr], $$

where $\eta(t) = \mathrm { e}^{-t}H(t)$, and *H* is the Heaviside step function. Equations (9)–(11) determine the interface dynamics for time-dependent spatially localised bump solutions that respect the Dirichlet boundary condition.

Since it is well known that the Amari model supports a stationary bump solution when the synaptic connectivity has a Mexican hat shape we now revisit this scenario and choose

12 $$ w(x) = \frac{1}{\sqrt{c \pi}} \biggl[\frac{a_{1}}{\sqrt{ b_{1}}} \mathrm { e}^{-x^{2}/b_{1}} - \frac{a_{2}}{\sqrt{ b_{2}}} \mathrm { e}^{-x^{2}/b_{2}} \biggr], $$

where $b_{1},b_{2},c >0$. Moreover, we will focus on the case that the stationary bump is symmetric about the origin. In this case demanding that the interface velocity is equal to zero requires that the numerator in (9) vanish. The formula for *ψ* given by (10) will also become time independent, and if we denote this by $\mathcal{P}(x)$ then we have

13 $$ \kappa= u_{\text{BC}} + \mathcal{P}(-\Delta/2)- \mathcal{P}(-L), $$

where we have set $x_{1} = -\Delta/2$ and $x_{2}=\Delta/2$ so that the *bump width* is given by $\Delta= x_{2}-x_{1}$. The formula for $\mathcal{P}$ is easily calculated as $\mathcal{P}(x) = p(x;a_{2},b_{2})-p(x;a_{1},b_{1})$, where

14 $$ p(x;a,b) = \frac{a}{2\sqrt{c}} \biggl[\mathrm{erf} \biggl(\frac {x_{1}-x}{\sqrt{b}} \biggr)-\mathrm{erf} \biggl(\frac{x_{2}-x}{\sqrt {b}} \biggr) \biggr]. $$

Hence, the bump width is determined implicitly by the single Eq. (13), and the bump shape, $q(x)$, is calculated from (6) as

15 $$ q(x) =u_{\text{BC}} + \mathcal{P}(x)- \mathcal{P}(-L). $$

To determine the stability of the bump solution we can follow the original approach of Amari and linearise the interface dynamics around the stationary values for $x_{i}$. Alternatively we can follow the Evans function approach, reviewed in [34], which considers perturbations at all values of *x* (rather than just at the bump edges). Here we pursue the latter approach, though it is straightforward to check that the former approach gives the same answer.

To determine the linear stability of a bump we write $u(x,t) = q(x) + \mathrm { e}^{\lambda t} \tilde{u}(x) $ where $\tilde{u} \ll 1$. In this case the corresponding change to *z* is given by $z(x,t) = \,\mathrm{d} q(x)/ \,\mathrm{d} x + \mathrm { e}^{\lambda t} \tilde{z}(x)$, where $\tilde{z}(x) = \partial\tilde{u}(x) /\partial x$. Expanding (5) to first order gives

16 $$ (\lambda+1) \frac {\mathrm {d} \tilde{u} (x)}{\mathrm {d} x} = \int_{-L}^{L} \,\mathrm{d} y w_{x} \bigl( \vert x-y \vert \bigr) \delta \bigl(q(y) -\kappa \bigr) \tilde{u} (y). $$

For the Dirac-delta function occurring under the integral, we can use the formal identity

17 $$ \delta \bigl(q(x) -\kappa \bigr) = \frac{\delta(x-x_{1})}{ \vert q'(x_{1}) \vert }+\frac {\delta(x-x_{2})}{ \vert q'(x_{2}) \vert }, $$

and integrate (16) from −*L* to *x* and use $\tilde {u}(-L)=0$ to obtain

18 $$\begin{aligned} (\lambda+1) \tilde{u}(x) ={}& \frac{\tilde{u}(x_{1})}{ \vert q'(x_{1}) \vert } \bigl[w \bigl( \vert x-x_{1} \vert \bigr) - w \bigl( \vert L+x_{1} \vert \bigr) \bigr] \\ & {}+ \frac{\tilde{u} (x_{2})}{ \vert q'(x_{2} )\vert } \bigl[w \bigl( \vert x-x_{2} \vert \bigr) -w \bigl( \vert L+x_{2} \vert \bigr) \bigr]. \end{aligned}$$

Here $q'(x)=\mathcal{P}'(x) = w( \vert x-x_{1} \vert )-w( \vert x-x_{2} \vert )$.

From (18) we may generate two equations for the amplitudes $(\tilde{u}(x_{1}),\tilde{u}(x_{2}))$ by setting $x=x_{1}$ and $x=x_{2}$. This gives a linear system of equations that we can write in the form $[\mathcal{A} -(\lambda+1) I](\tilde{u}(x_{1}),\tilde {u}(x_{2}))=(0,0)$, where

19 $$ \mathcal{A} = \begin{bmatrix} \frac{w(0)-w(L+x_{1})}{ \vert q'(x_{1}) \vert } & \frac{w(\Delta )-w(L+x_{2})}{ \vert q'(x_{2}) \vert }\vphantom{\displaystyle\max_{1}} \\ \frac{w(\Delta)-w(L+x_{1})}{ \vert q'(x_{1}) \vert } & \frac {w(0)-w(L+x_{2})}{ \vert q'(x_{2}) \vert } \end{bmatrix}. $$

Requiring non-trivial solutions gives a formula for the spectrum as $\det[\mathcal{A} -(\lambda+1) I]=0$, which yields

20 $$ \lambda_{\pm}= -1 + \frac{\operatorname{Tr} \mathcal{A} \pm\sqrt {(\operatorname{Tr} \mathcal{A})^{2} - 4 \det\mathcal{A}}}{2}. $$

Hence a bump solution will be stable provided $\text{Re} \lambda_{\pm}<0$.

**Fig. 1:**
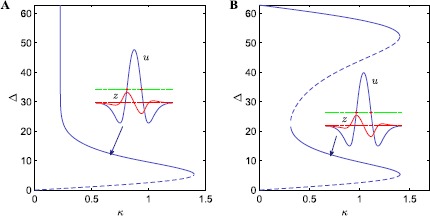
Effect of Dirichlet boundary condition $u_{BC} = 0$ on the bifurcation diagram of a bump solution. (**A**): bump solutions for Eq. (1) with $\varOmega= [-10\pi, 10 \pi]$. (**B**): bumps solutions for Eqs. (3)–(4) posed on $\varOmega= [-10\pi, 10 \pi]$ with Dirichlet boundary condition $u_{BC} = 0$. Stable (unstable) solutions are indicated with solid (dashed) lines. The insets show the shapes of the lower (stable) bumps at $\kappa= 0.7$ for $q(x)$ (blue) and $z(x)$ (red). Parameters are $a_{1} =14$, $a_{2} = 13$, $b_{1} = 24$, $b_{2} = 150$, $c=5$

In Fig. 1 we plot the bump width as a function of the threshold *κ* for a neural field posed on a finite domain Fig. 1(A) and, for the reformulated neural field with an imposed Dirichlet boundary condition $u_{\mathrm{BC}}=0$ Fig. 1(B), using solid (dashed) lines for stable (unstable) solutions. For the former case we recover the expected Amari result, namely that there is coexistence between two bumps, the widest of which is stable. However, when we impose a Dirichlet boundary condition, four coexisting bumps are found for sufficiently large *κ*, and two of these bumps are stable. In other words, the Dirichlet boundary condition induces a new stable bump, whose active region occupies a large portion of the domain.

## Two Spatial Dimensions: Infinite Domain

Before discussing the extension of Sect. 3 to a finite two-dimensional domain with an imposed boundary condition it is first instructive to consider the problem posed on $\mathbb{R}^{2}$. An interface description for this case was originally developed in [21], albeit for a special choice of synaptic connectivity kernel. By exploiting certain properties of the modified Bessel function of the second kind it was possible to reformulate integrals over two-dimensional domains in terms of one-dimensional line integrals. This allowed the interface dynamics to be expressed solely in terms of the shape of the active region in the tissue, namely a one-dimensional closed curve. Here we extend this approach to a far more general class of synaptic connectivity kernels, which include combinations of radially symmetric Gaussian functions (12).

**Fig. 2:**
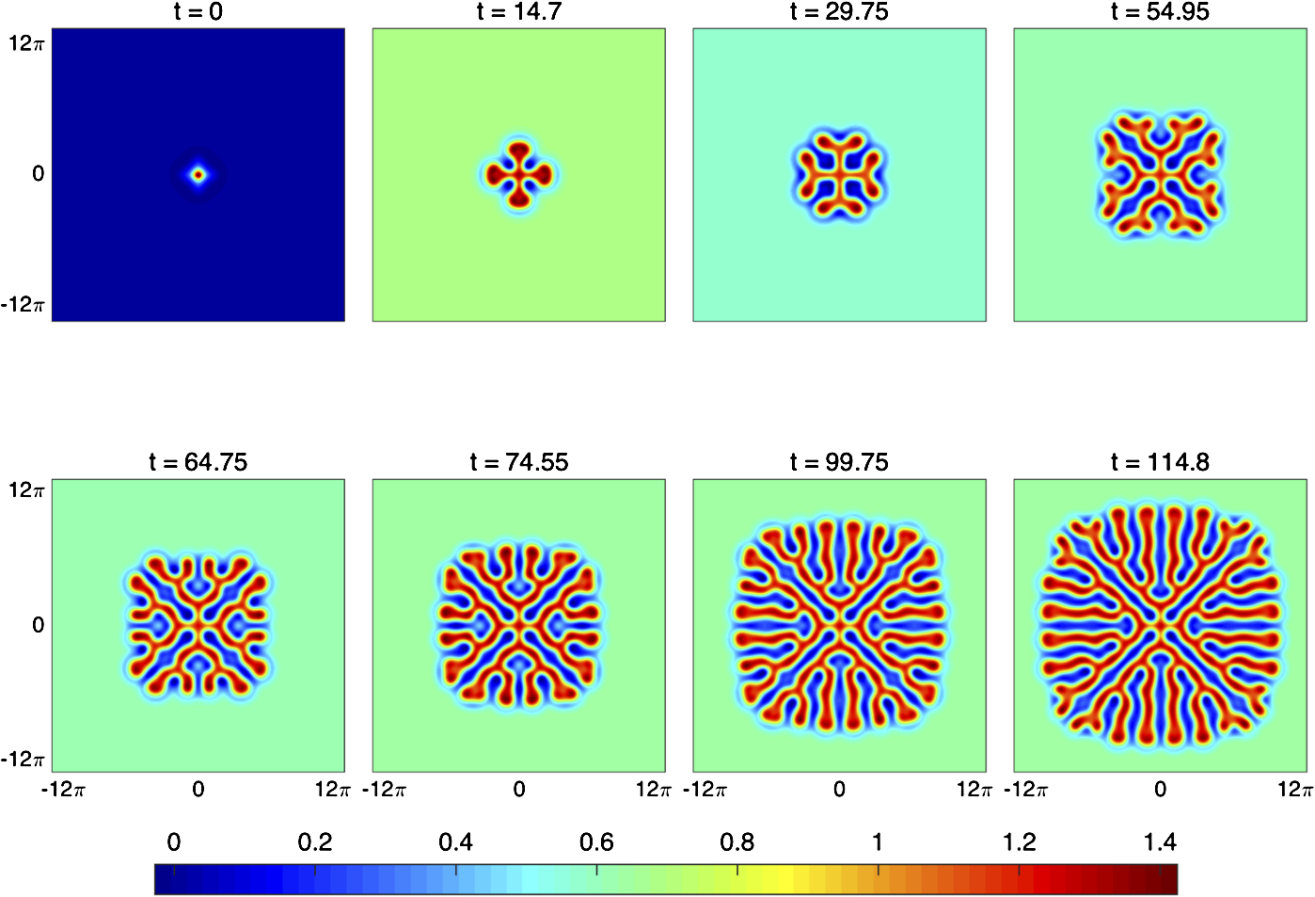
Space-time simulations of the field $u(\boldsymbol {x},t)$ showing a spreading labyrinthine structure in a 2D Amari model (on a large domain $[-L,L] \times[-L,L]$) with a radially symmetric difference of Gaussians connectivity, namely $w(\boldsymbol {r})=w(r)$, with $w(r)$ given by (12). Parameters are $\kappa= 0.03$, $a_{1} =3.55$, $a_{2} = 3$, $b_{1} = 2.4$, $b_{2} = 3.2$, $c=10$, and $L=12\pi$

We consider the integro-differential equation given by (1) with $\varOmega= \mathbb{R}^{2}$. We decompose the domain *Ω* by writing $\varOmega= \varOmega_{+} \cup\partial\varOmega_{+} \cup \varOmega_{-}$ where $\partial\varOmega_{+}$ represents the level-set which separates $\varOmega_{+}$ (excited) and $\varOmega_{-}$(quiescent) regions. These domains are given explicitly by $\varOmega_{+} = \{ \boldsymbol {x} | u(\boldsymbol {x}) >\kappa\}$, $\varOmega_{-} = \{ \boldsymbol {x} | u(\boldsymbol {x}) <\kappa\}$, and $\partial\varOmega_{+} = \{ \boldsymbol {x} | u(\boldsymbol {x}) =\kappa\}$. We shall assume that $\partial\varOmega_{+}$ is a closed contour (or a finite set of disconnected closed contours). In Fig. 2 we show a direct numerical simulation of the full space–time model to illustrate that a synaptic connectivity function that is a radially symmetric difference of Gaussians can support a spreading labyrinthine pattern. Similar patterns have previously been reported and discussed in [21, 35] for both Heaviside and steep sigmoidal firing rate functions. A description of the numerical scheme used to evolve the full space–time model is given in Appendix 1. Differentiation of the level-set condition $u(\partial\varOmega_{+}(t),t)=\kappa$ gives the normal velocity rule:

21 $$ c_{n} \equiv{ \boldsymbol {n}}\cdot \frac {\mathrm {d} }{\mathrm {d} t} \partial\varOmega_{+} = \frac {u_{t}(\boldsymbol {x},t)}{\nabla_{\boldsymbol {x}} u(\boldsymbol {x},t)} \Big| _{\boldsymbol {x}=\partial\varOmega_{+}(t)}, $$

where we have introduced the normal vector $\boldsymbol {n} = - \nabla_{\boldsymbol {x}} u / \vert \nabla_{\boldsymbol {x}} u \vert $ along $\partial\varOmega_{+}(t)$. We will now show that $c_{n}$ can be expressed solely in terms of integrals along $\partial\varOmega_{+}(t)$. Let us first consider the denominator in (21). The temporal integration of (4), using variation of parameters, gives

22 $$ \nabla_{\boldsymbol {x}} u (\boldsymbol {x},t) = z(\boldsymbol {x},t) = \eta(t) z_{0}( \boldsymbol {x}) + \int_{0}^{t} \,\mathrm{d} t' \eta \bigl(t' \bigr) \nabla_{\boldsymbol {x}} \psi \bigl( \boldsymbol {x},t-t' \bigr), $$

where $\eta(t) = \mathrm { e}^{-t} H(t)$, $z_{0}(\boldsymbol {x})=\nabla_{\boldsymbol {x}} u(\boldsymbol {x},0)$ denotes gradient information at $t=0$, and

23 $$ \psi(\boldsymbol {x},t) = \int_{\varOmega_{+}(t)}\,\mathrm{d}\boldsymbol {y} w \bigl( \vert \boldsymbol {x}-\boldsymbol {y} \vert \bigr). $$

The term $\nabla_{\boldsymbol {x}} \psi$ in (22) can be constructed as a line integral using the integral vector identity:

24 $$ \nabla_{\boldsymbol {x}} \psi(\boldsymbol {x},t) = \int_{\varOmega_{+}(t)} \,\mathrm{d}\boldsymbol {y} \nabla_{\boldsymbol {x}}w \bigl( \vert \boldsymbol {x}-\boldsymbol {y} \vert \bigr) = - \oint_{\partial\varOmega _{+}(t)} \,\mathrm{d} s \boldsymbol {n}(s) w \bigl( \bigl\vert \boldsymbol {x}- \boldsymbol {y}(s) \bigr\vert \bigr). $$

Thus the denominator in (21) can be expressed solely in terms of a line integral around the contour $\partial\varOmega_{+}(t)$. The representation of the numerator in (21) in terms of a line integral rather than a double integral is more challenging. In previous work we have shown that this can be achieved for the special case that the weight kernel is constructed from a linear combination of zeroth order modified Bessel functions of the second kind [21]. In Appendix 2 we show that a line-integral representation can be constructed for a far more general class of anatomical connectivity patterns, making use of the divergence theorem. Using this result the numerator of (21) can be written

25 $$ u_{t} \bigl(\partial\varOmega_{+}(t),t \bigr) = -\kappa+ \psi \bigl( \partial\varOmega_{+}(t),t \bigr), $$

where

26 $$ \psi(\boldsymbol {x},t) = \oint_{\partial\varOmega_{+}(t)} \,\mathrm{d} s \varphi \bigl( \bigl\vert \boldsymbol {x}- \boldsymbol{\gamma}(s) \bigr\vert \bigr)\frac{\boldsymbol {x}-\boldsymbol{\gamma}(s)}{ \vert \boldsymbol {x}-\boldsymbol{\gamma}(s) \vert } \cdot \boldsymbol {n}(s) + \mathcal {K} C, $$

and *s* is a parametrisation for points on the contour $\boldsymbol {\gamma} \in\partial\varOmega_{+}$. Here,

27 $$\begin{aligned} \varphi(r)&=\frac{1}{r} \int_{\infty}^{r} x w(x) \,\mathrm{d} x, \qquad \mathcal{K}= \int_{\mathbb{R}^{2}} w(\boldsymbol {x}) \,\mathrm{d}\boldsymbol {x}\quad \text{and} \\ C &= \textstyle\begin{cases} 1 & \text{if $\boldsymbol {x} \in\varOmega_{+}$}, \\ 1/2 & \text{if $\boldsymbol {x} \in\partial\varOmega_{+}$},\\ 0 & \text{if $\boldsymbol {x} \in\varOmega_{-}$}. \end{cases}\displaystyle \end{aligned}$$

Hence the normal velocity rule (21) can be expressed solely in terms of one-dimensional line integrals involving the shape of the active region $\varOmega_{+}$ (which is prescribed by $\partial\varOmega_{+}$). This is a substantial reduction in description as compared to the full space–time model, yet is exact.

As an example of the approach above let us consider a difference of Gaussians with $w(r)$ given by (12). A simple calculation for this choice shows that $\mathcal{K}= \sqrt{\pi/c} [a_{1} \sqrt{b_{1}} - a_{2} \sqrt{b_{2}}]$ and

28 $$ \varphi(r) = \frac{1}{2 r \sqrt{c \pi}} \bigl[a_{2} \sqrt{b_{2}} \mathrm { e}^{-r^{2}/b_{2}}-a_{1} \sqrt{b_{1}} \mathrm { e}^{-r^{2}/b_{1}} \bigr] . $$

**Fig. 3:**
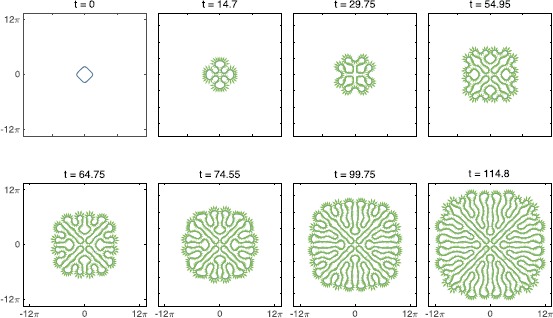
A numerical simulation of the interface dynamics for the same scenario as Fig. 2. Here the threshold condition where $u=\kappa$ is given by the solid blue line, whilst the green arrows show the normal velocity of the moving interface. All parameters as in Fig. 2

In Fig. 3 we show a numerical simulation prescribed by the interface method, with initial data equivalent to that from the full space–time simulation shown in Fig. 2. The excellent agreement between the two figures is easily observed. The full details of our numerical scheme for implementing the interface dynamics are given in Appendix 3.

## Two Spatial Dimensions: Dirichlet Boundary Condition

Using the notation of Sect. 4 we now show how to extend the one-dimensional approach of Sect. 3 to develop an interface dynamics for planar Amari models on a bounded domain with Dirichlet boundary conditions. For a single active region the dynamics for $\boldsymbol {z}(x,t)$ is given by (4), which can be written succinctly as

29 $$ \frac {\partial \boldsymbol {z}(\boldsymbol {x},t)}{\partial t} = -\boldsymbol {z}(\boldsymbol {x},t) + \nabla_{\boldsymbol {x}} \psi( \boldsymbol {x},t), $$

with *ψ* given by (23), or in terms of $\partial \varOmega_{+}(t)$, by (26). Using (3) the level-set condition is

30 $$ \kappa-u_{\text{BC}} = \int_{\varGamma(\partial\varOmega_{+}(t))} \boldsymbol {z}(\boldsymbol {r},t)\cdot\,\mathrm{d}\boldsymbol {r}. $$

Using the identity

31 $$ \frac {\mathrm {d} }{\mathrm {d} t} \int_{\varGamma(\partial\varOmega_{+}(t))} z(\boldsymbol {r},t)\cdot\,\mathrm{d} \boldsymbol {r} = \boldsymbol {z} \bigl(\partial \varOmega_{+}(t),t \bigr) \cdot \frac {\mathrm {d} }{\mathrm {d} t} \partial\varOmega _{+}(t) + \int_{\varGamma(\partial\varOmega_{+}(t))} z_{t}(\boldsymbol {r},t)\cdot \,\mathrm{d}\boldsymbol {r}, $$

and differentiating (30) with respect to *t*, we obtain the normal velocity rule

32 $$ c_{n} \equiv{ \boldsymbol {n}}\cdot \frac {\mathrm {d} }{\mathrm {d} t} \partial\varOmega_{+} = \frac{1}{ \vert \boldsymbol {z}(\boldsymbol {x},t) \vert } \int_{\varGamma(\boldsymbol {x})} \boldsymbol {z}_{t}(\boldsymbol {r},t)\cdot\,\mathrm{d} \boldsymbol {r} \Big\vert _{\boldsymbol {x}=\partial\varOmega_{+}(t)}. $$

Here the normal vector is given by $\boldsymbol {n} = -\boldsymbol {z}/ \vert \boldsymbol {z} \vert $ along the contour $\partial\varOmega_{+}$. Using (29) we may write the numerator in the normal velocity rule (32) as

33 $$ \int_{\varGamma(\partial\varOmega_{+}(t))} \boldsymbol {z}_{t}(\boldsymbol {r},t)\cdot\,\mathrm{d} \boldsymbol {r} = u_{\text{BC}}-\kappa+\psi \bigl(\partial\varOmega_{+}(t),t \bigr) -\psi \bigl( \boldsymbol {\zeta} \bigl(\partial\varOmega_{+}(t) \bigr),t \bigr), $$

where $\boldsymbol {\zeta}: \partial\varOmega_{+}(t) \rightarrow\partial\varOmega $ is a mapping from points on the contour $\partial\varOmega_{+}(t)$ to points on the boundary *∂Ω*.

Hence, using the formulae for ***z*** and *ψ* from Sect. 4, namely Eqs. (22), (24), and (26), then all of the terms in the normal velocity rule (32) may be expressed as one-dimensional line integrals. This yields the interface dynamics for Dirichlet boundary conditions, and once again we see that it is a reduced yet exact alternative formulation to the full space–time model. In contrast to the interface dynamics on an infinite domain one needs only develop further numerical algorithms for computing the line integral in (3). The numerical method for implementing the interface dynamics can be based upon that, for an infinite domain, with a specific choice for the paths *Γ* defining this integral. Each of the paths *Γ* connects a point ***x*** in the interior of the domain to a point on the boundary, and we set $\boldsymbol {\zeta}(\partial\varOmega_{+}(t))$ to be the endpoint of $\varGamma (\partial\varOmega_{+}(t))$ (see Appendix 3 for details on the numerical scheme).

Note that we do not have to numerically integrate along this path (to determine the normal velocity), and that we need only to determine the values of $\psi(\boldsymbol {x},t)$ at the two endpoints.

**Fig. 4:**
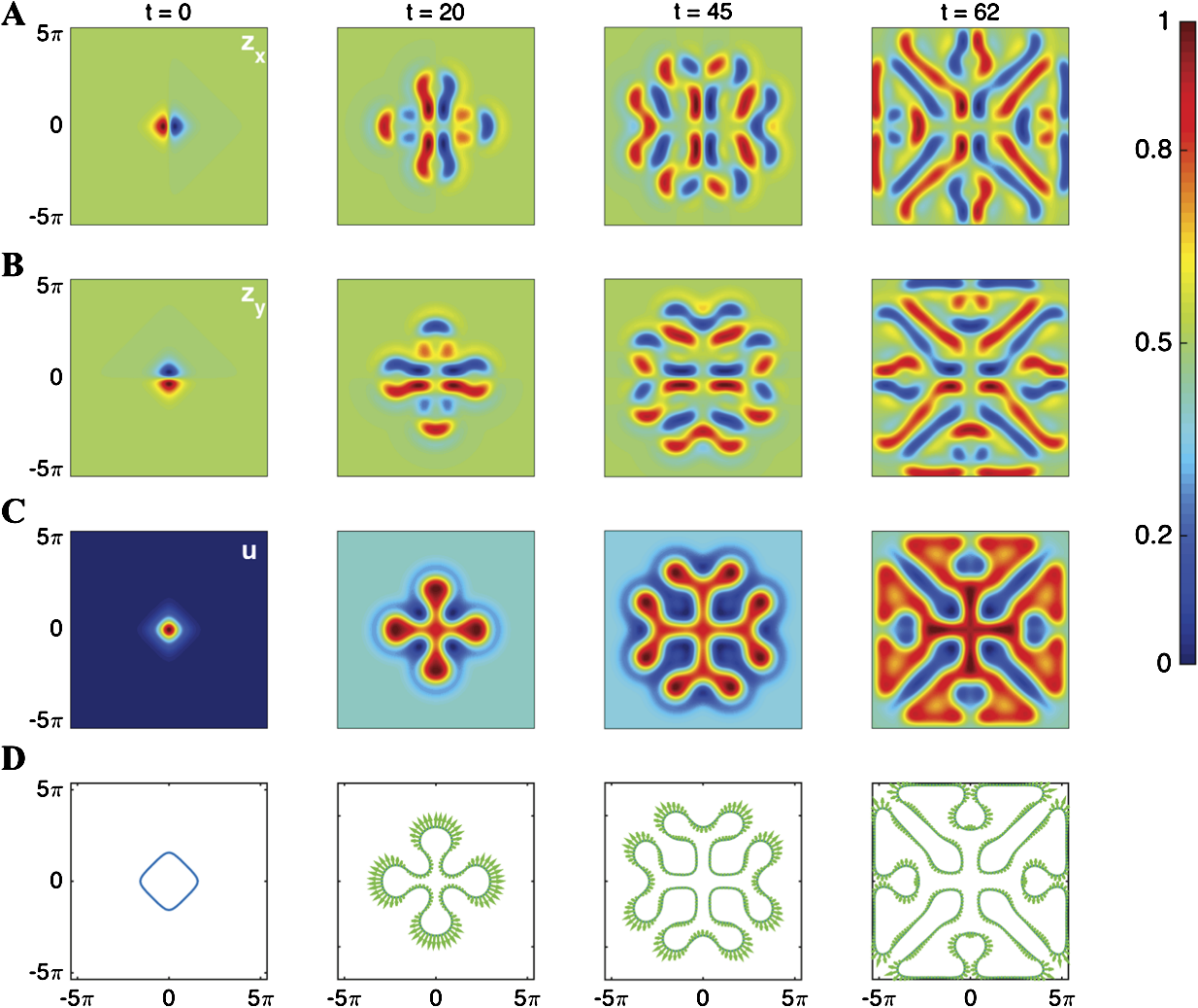
A spreading pattern (**C**) governed by the space–time model (3) and (4) with a radially symmetric synaptic connectivity kernel given by (12) and a Dirichlet boundary condition $u_{\text{BC}}=0$ on a domain of size $[-L,L] \times [-L,L]$. The corresponding interface dynamics is shown in (**D**). Rows (**A**) and (**B**) show the components of the gradient ***z*** in the *x* and *y* directions, and these are used to compute the activity of the neuronal tissue shown in row (**C**). Parameters are $\kappa=0.05$, $a_{1} =3.55$, $a_{2} = 3$, $b_{1} = 2.4$, $b_{2} = 3.2$, $c=10$, and $L=5\pi$

Figure 4 shows a direct numerical simulation computed using the evolution of the gradient $\boldsymbol {z}= \nabla_{\boldsymbol {x}} u$ as well as the corresponding interface dynamics. We see excellent agreement between the two approaches. The obvious advantage of the interface dynamics is that one need only evolve the shape of the active region to fully reconstruct the full space–time dynamics using (3) and (24). We see from Fig. 4 that the main effect of the Dirichlet boundary condition is to limit the spread of a labyrinthine structure and ultimately induce a highly structured stationary pattern, as expected.

## Spots in a Circular Domain: Dirichlet Boundary Condition

Given the large amount of historical interest in spot solutions of neural field models on infinite domains, and those on finite domains without incorporating the role of boundary conditions [35–39], it is worthwhile to revisit this specific class of solutions on a finite disc with an imposed Dirichlet boundary condition. We shall consider radially symmetric synaptic connectivity kernels and a disc of radius *D* with a spot (circularly symmetric) solution of radius *R*. In this case $u(\boldsymbol {r},t)=q(r)$ with $r= \vert \boldsymbol {r} \vert $ for all *t*, and $q(D)=u_{\text{BC}}$, with $q(R)=\kappa$ and $q(r) > \kappa $ for $r< R$ and $q(r) < \kappa$ for $R< r< D$. We shall denote the corresponding stationary field for *ψ* by $\psi (r)$, and this is conveniently constructed from (26).

### Construction

An implicit equation for the radius of the bump is obtained after setting the normal velocity to zero. Using (32) and (33) this yields

34 $$ \kappa= u_{\text{BC}} + \psi(R) - \psi(D). $$

For the specific choice of a difference of Gaussians given by (12) we may write this in the form

35 $$ \psi(r) = \frac{a_{1}}{\sqrt{c \pi b_{1}}} \rho(r;b_{1})-\frac {a_{2}}{\sqrt{c \pi b_{2}}} \rho(r;b_{2}) +\mathcal{K} C, $$

with

36 $$ \rho(r;\alpha) = - \frac{\alpha}{2} \int_{0}^{2\pi} \,\mathrm{d}\theta \frac{\mathrm { e}^{-\mathcal{Q}(\theta)^{2}/\alpha}}{\mathcal{Q}(\theta )^{2}} R (r\cos \theta-R), $$

and $\mathcal{Q}(\theta) = \sqrt{R^{2} + r^{2} -2 R r \cos\theta}$. Although (36) is in closed form it is a challenge to perform the integral analytically. Thus it is also of interest to consider synaptic connectivity kernels for which more explicit progress can be made. A case in point is that of piece-wise constant functions.

Let us first consider a top-hat connectivity defined by

37 $$ w(r) = \textstyle\begin{cases} w_{+} >0, & r \leq\sigma, \\ w_{-} < 0, & r > \sigma. \end{cases} $$

In this case it is easier to construct $\psi(r)$ directly from (23) as

38 $$ \psi(r) = \int_{ \vert \boldsymbol {r}' \vert < R} \,\mathrm{d}\boldsymbol {r}' w \bigl( \bigl\vert \boldsymbol {r}-\boldsymbol {r}' \bigr\vert \bigr). $$

For the top-hat shape (37) we may split the above integral as

39 $$ \psi(r) = w_{+} \mathop{ \int_{ \vert \boldsymbol {r}' \vert < R}}_{ \vert \boldsymbol {r}-\boldsymbol {r}' \vert \leq\sigma} \,\mathrm{d}\boldsymbol {r}' + w_{-} \mathop{ \int_{ \vert \boldsymbol {r}' \vert < R}}_{ \vert \boldsymbol {r}-\boldsymbol {r}' \vert > \sigma} \,\mathrm{d}\boldsymbol {r}'. $$

Introducing the area $A_{+}(r,\sigma)$ as

40 $$ A_{+}(r, \sigma) = \mathop{ \int_{ \vert \boldsymbol {r}' \vert < R}}_{ \vert \boldsymbol {r}-\boldsymbol {r}' \vert \leq\sigma} \,\mathrm{d}\boldsymbol {r}',\quad r= \vert \boldsymbol {r} \vert , $$

where $A_{+}(R, \sigma) = \kappa$, the self-consistent equation for a spot (34) takes the form

41 $$ \kappa=u_{BC}+ (w_{+} - w_{-}) \bigl[ A_{+}(R, \sigma) - A_{+}(D, \sigma) \bigr]. $$

Following the work of Herrmann *et al*. [40], we now show how to evaluate the integral (40) using simple geometric ideas. For example, the area $A_{+}(R, \sigma)$ can be calculated in terms of the area of overlap of two circles, one of centre **0** and radius $\vert \boldsymbol {r} \vert=R$, and the other of centre ***r*** and radius *σ* subject to the constraint $r=R$.

Using the results from Appendix 4 we have $A_{+}(r, \sigma)=A(R,\phi_{0}(r,\sigma))+A(\sigma,\phi_{1}(r,\sigma ))$, where $A(r,\phi) = r^{2}(\phi-\sin\phi)/2$ and

42 $$\begin{aligned} \phi_{0}(r,\sigma)&= 2 \cos^{-1} \biggl( \frac{ R^{2}-\sigma^{2} +r^{2}}{2 R r} \biggr), \\ \phi_{1}(r,\sigma)&= 2 \cos^{-1} \biggl( \frac{\sigma^{2}- R^{2} +r^{2}}{2 \sigma r} \biggr), \end{aligned}$$

with $R>D-\sigma$.

Another natural piece-wise constant choice is the piece-wise constant Mexican hat shape given by

43 $$ w(r) = \textstyle\begin{cases} w_{+} >0, & r \leq\sigma_{1} \\ w_{-} < 0, & \sigma_{1} < r \leq\sigma_{2}\\ 0, & r>\sigma_{2} \end{cases}\displaystyle ,\quad \sigma_{2} > \sigma_{1} . $$

Using a similar argument to that for the top-hat connectivity we find that

44 $$\begin{aligned} \kappa={}&u_{BC} + (w_{+}- w_{-}) \bigl[ A_{+}(R, \sigma_{1}) - A_{+}(D,\sigma _{1}) \bigr] \\ &{}+ w_{-} \bigl[ A_{+}(R, \sigma_{2}) - A_{+}(D,\sigma_{2}) \bigr], \end{aligned}$$

with $R>D-\sigma_{1}$.

### Stability

The stability of spots without boundary conditions has been treated by several authors, and see [38] for a recent overview. Here we extend this approach to treat a finite domain with an imposed Dirichlet boundary condition following very similar arguments to those presented in Sect. 3.

To determine the linear stability of a spot we write $u(\boldsymbol {r},t) = q(r) + \mathrm { e}^{\lambda t} \cos(m \theta) \tilde{u}(r) $ where $\tilde{u} \ll1$ and $m\in \mathbb{N}$. In this case the corresponding change to ***z*** is given by $\boldsymbol {z}(\boldsymbol {r},t) = \nabla_{\boldsymbol {r}} q(r)+ \mathrm { e}^{\lambda t} \cos(m \theta) \tilde{ \boldsymbol {z}}(\boldsymbol {r})$, where $\tilde{\boldsymbol {z}}(\boldsymbol {r})= \nabla_{\boldsymbol {r}} \tilde{u}(r)$. Expanding (4) to first order gives

45 $$ (\lambda+1) \tilde{\boldsymbol {z}} (\boldsymbol {r}) = \int_{0}^{2 \pi} \,\mathrm{d}\theta \cos(m \theta) \int_{0}^{\infty}r' \,\mathrm{d} r' \nabla_{\boldsymbol {r}} w \bigl( \bigl\vert \boldsymbol {r}- \boldsymbol {r}' \bigr\vert \bigr) \delta \bigl(q \bigl(r' \bigr)-\kappa \bigr) \tilde{u} \bigl(r' \bigr), $$

where $\vert \boldsymbol {r}-\boldsymbol {r}' \vert =\sqrt{r^{2}+r^{\prime 2} -2 r r' \cos\theta}$. Using properties of the Dirac-delta distribution we find

46 $$ \nabla_{\boldsymbol {r}} \biggl[ (\lambda+1) \tilde{u}(r) - \tilde{u}(R) \frac{R}{ \vert q'(R) \vert } \int _{0}^{2 \pi} \,\mathrm{d}\theta\cos(m \theta) w \bigl( \bigl\vert \boldsymbol {r}-\boldsymbol {r}' \bigr\vert \bigr) |_{r'=R} \biggr]=0. $$

Since the term in square brackets in (46) is radially symmetric we may integrate in the radial direction using $\tilde{u}(D)=0$ to obtain

47 $$\begin{aligned} &(\lambda+1) \frac{\tilde{u}(r)}{\tilde{u}(R)} \\ &\quad= \frac{R}{ \vert q'(R) \vert } \int_{0}^{2 \pi} \,\mathrm{d}\theta\cos(m \theta) \bigl[ w \bigl( \bigl\vert \boldsymbol {r}-\boldsymbol {r}' \bigr\vert \bigr)| _{r'=R} - w \bigl( \bigl\vert \boldsymbol {r}-\boldsymbol {r}' \bigr\vert \bigr) | _{{r'=R, r=D}} \bigr]. \end{aligned}$$

Setting $r=R$ in (47) and demanding non-trivial solutions gives an equation for the eigenvalues *λ* in the form $\mathcal{E}_{m}(\lambda)=0$, $m \in \mathbb{N}$, where

48 $$\begin{aligned} \mathcal{E}_{m}(\lambda) = {}&\lambda+1 - \frac{R}{ \vert q'(R) \vert } \int_{0}^{2 \pi} \,\mathrm{d}\theta\cos(m \theta) \bigl[ w \bigl( \bigl\vert \boldsymbol {r}-\boldsymbol {r}' \bigr\vert \bigr) | _{{r'=R, r=R}} \\ &{}- w \bigl( \bigl\vert \boldsymbol {r}-\boldsymbol {r}' \bigr\vert \bigr) | _{{r'=R,r=D}} \bigr]. \end{aligned}$$

Thus a spot solution will be stable provided $\lambda_{m} <0$ for all $m \in \mathbb{N}$ where $\lambda_{m}$ is a zero of $\mathcal{E}_{m}(\lambda)$. Once again the choice of a piece-wise constant connectivity function considerably simplifies further calculations. For example for the top-hat function given by (37) it is simple to show that

49 $$ q'(R) = \frac{\sigma (w_{-}-w_{+}) }{R}\sqrt{4 R^{2}- \sigma^{2}}, $$

and

50 $$ \int_{0}^{2 \pi} \,\mathrm{d}\theta\cos(m \theta) w \bigl( \bigl\vert \boldsymbol {r}-\boldsymbol {r}' \bigr\vert \bigr)| _{{r'=r=R}} = 2 \biggl( \frac{w_{+}-w_{-}}{m} \biggr) \sin m \theta^{*}, $$

where $\theta^{*}$ is the smaller of the two roots of the equation $R \sqrt{2(1-\cos\theta)}=\sigma$ for $\theta\in[0,2 \pi)$. Equation (50) allows for the explicit evaluation of (48) for a piece-wise constant synaptic connectivity.

**Fig. 5:**
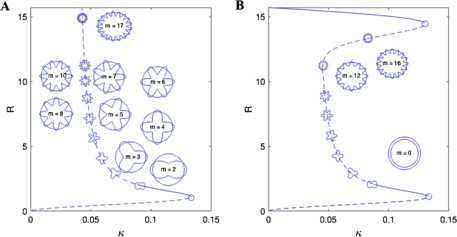
Spot radius *R* as a function of *κ* for a smooth Mexican hat connectivity given by (12), with parameters as in Fig. 4. (**A**): Infinite domain. (**B**): Finite domain that is a disc of radius $D =5 \pi$, with Dirichlet boundary condition $q(D)=u_{\text{BC}}=0$. Linear stability analysis shows that solid (dashed) lines are stable (unstable). Azimuthal instabilities with various modes are indicated by the mode shapes

Using the above analysis we find that, for the smooth Mexican hat function, given by (12), that for large domains a wide and narrow spot can coexist for a sufficiently low value of the threshold *κ*. Moreover, the narrow spots are always unstable (to modes with $m=0$, reflecting uniform changes of size), whilst the wider spots can develop instabilities to modes with $m \geq2$. We note that the mode with $m=1$ is always expected to exist due to rotational invariance (and would give rise to a zero eigenvalue for all parameter values). This is entirely consistent with previous results for Mexican hat connectivities on domains where no boundary condition is used, as reviewed in [38]. However, on a finite size disc and with an imposed Dirichlet boundary condition further spots can be induced, with sizes commensurate to that of the radius of the disc. Both of these scenarios are summarised with the use of Fig. 5. Qualitatively similar behaviour is found for the piece-wise constant Mexican hat function given by (43) (not shown). Interestingly for the simple top-hat connectivity, given by (37), we find similar results for existence, though without azimuthal instabilities to modes $m \geq2$.

## Discussion

In this paper we have revisited the seminal work of Amari on neural fields and shown how to incorporate Dirichlet boundary conditions. We have built on the previous work of Coombes *et al*. [21] to develop an interface dynamics approach for the evolution of closed curves defining pattern boundaries. Compared to the full space–time model with imposed Dirichlet boundary conditions the interface dynamics is reduced, yet requires no approximations. The interface framework has been illustrated in a number of settings in both one- and two-dimensions, with a focus on localised states and their instabilities. In all cases we have highlighted the excellent correspondence between results obtained from numerical simulations of the full space–time model and the interface approach. Moreover, we have also emphasised that for piece-wise constant synaptic connectivities the interface approach becomes quasi-analytical, in that many of the terms required for the computation of the normal velocity of the interface can be calculated by hand rather than have to be found numerically. For spreading patterns that may arise from the azimuthal instability of a localised spot, the main effect of a Dirichlet boundary condition has been to limit the growth of the pattern. This was entirely expected, although the precise shape of the resulting stationary pattern is of course hard to predict without simulation. However, the induction of other branches of localised states in a neural field model on a disc was more surprising, even though all near the boundary proved to be stable. It should be noted that the imposition of different boundary conditions may effect the spatio-temporal evolution of a pattern and the conditions for its dynamics instability. For the sake of computational simplicity, the value attained by the activity variable at the boundary was chosen to be a constant ($u_{BC}=0$) throughout this paper. However, a full analysis which also treats space- and time-dependent boundary conditions can be readily developed for the direct numerical simulations, as well as for the equivalent interface description. There are a number of natural extensions of the approach that we have presented here to treat other, more biophysically rich, neural field models which we outline below.

Although we have focussed exclusively on the Amari model with a Heaviside firing rate, direct numerical simulations (not shown) readily confirm that boundary induced patterns can also be seen in models with a smooth sigmoidal firing rate. Thus it would also be of interest to extend the elegant functional analytic treatment of localised states in bounded domains by Faugeras *et al*. [14] to incorporate imposed boundary conditions. Some form of spike frequency adaptation (SFA) is often included in neural field models to mimic a negative feedback process to diminish sustained firing. This can cause a travelling front to transition to a travelling pulse [41], or subserve the generation of planar spiral waves [28]. If this current is linear, as is often the case [42], or itself described by dynamics involving a Heaviside switch, as in [43], then the interface approach presented here can be generalised. Given previous work on equivalent PDE models on bounded domains with SFA that analyses spiral wave behaviour, the treatment of spiral waves from an interface perspective would be an advance as it is not limited to synaptic connectivities with a rational Fourier structure [44]. Another natural extension of the work in this paper is to neural fields on *feature spaces*. For example, in the primary visual cortex (V1), cells respond preferentially to lines and edges of a particular orientation. A standard neural field model that links points at ***r*** and $\boldsymbol {r}'$ (in the plane) with a weight $w(\boldsymbol {r}|\boldsymbol {r}')$, should be replaced by a more general form such as $w(\boldsymbol {r}|\boldsymbol {r}')=w(\boldsymbol {r},\theta|\boldsymbol {r}',\theta')$, where *θ* ($\theta'$) would represent an orientation preference at ***r*** ($\boldsymbol {r}'$). This model has recently been studied using a neural field dynamics with a Heaviside firing rate [45], and is thus ripe for a further analysis using an interface approach. Finally it is worth pointing out the rather pertinent difference between the flat models we have discussed here and the well-known folded characteristic of real cortex, with its sulci and gyri. Fortunately there is no substantial difficulty in formulating neural field models on curved surfaces, though to date there has been surprisingly little analysis of spatio-temporal pattern formation in this context. The exception to this rule is the simulation studies of Bojak *et al*. [46], and the recent work of Sato *et al*. for growing brains [13].

One obvious caveat to all of the above is that the interface approach is restricted to Amari style models with a Heaviside firing rate. Nonetheless the qualitative similarities between Amari models and those with a steep sigmoidal firing rate are well known. In summary the treatment of neural fields with boundary conditions is a relatively unexplored area of mathematical neuroscience whose further study should pay dividends for the understanding of neuroimaging data, and in particular waves of activity in functionally identified and folded cortices.

## Figures and Tables

**Fig. 6:**
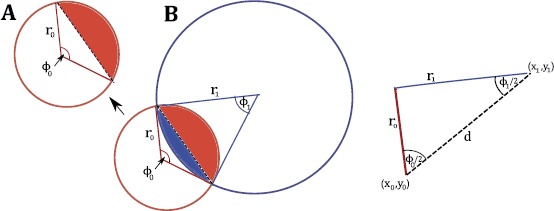
The area of the total shaded segment is $r_{0}^{2} (\phi_{0} -\sin \phi_{0})/2$ (**A**). Overlap of two circles shows the area of active region (**B**)

## Appendix 1: Numerical Scheme for the Full Space-Time Model

The numerical simulation of the full space–time model (1) without boundary conditions was performed by discretising the domain on an *N*-by-*N* tensor grid, and using a Nyström scheme for the spatial discretisation [47]. It is known that the major costs of this scheme is in the evaluation of the Hammerstein operator occurring on the right-hand side of (1), which on the grid outlined above requires $N^{4}$ operations. Owing to the convolutional structure of the operator, it is possible to decrease considerably the computational cost of each function evaluation by performing a pseudo-spectral evaluation of the convolution, using a Fast Fourier Transform (FFT), followed by an inverse Fast Fourier Transform (IFFT). This reduces the number of operations to $O(N^{2} \log N^{2})$ and allows one to simulate neural fields and compute equilibria efficiently. We refer the reader to [16, 21] for further details. In these calculations we set $N=2^{9}$ and used Matlab’s in-built ode45 routine, with standard tolerance settings.

In simulations where the state variable is not periodic, such as the ones where we enforced boundary conditions (Sect. 5) we used a standard matrix-vector multiplication to evaluate the integral operator. A full matrix was precomputed and stored during the initialisation phase of the time stepping and the grid-size was limited to $N=2^{7}$ points, owing to memory constraints. In this setting, the interface dynamic approach becomes a viable alternative to the full spatial simulation.

## Appendix 2: Expressing *ψ* in Terms of Contour Integrals

In this appendix, we derive the identities (26) and (27). This allows us to represent the double integral for the non-local input $\psi(\boldsymbol {x},t)$ given by (23) as an equivalent line integral. We recall divergence theorem for a generic vector field ***F*** on a domain $\mathcal{B}$ with boundary $\partial\mathcal{B}$,

51 $$ \int_{\mathcal{B}} (\nabla\cdot \boldsymbol {F} ) \,\mathrm{d}\boldsymbol {x} = \oint_{\partial\mathcal{B}} \boldsymbol {F}\cdot \boldsymbol {n} \,\mathrm{d} s, $$

where ***n*** is the unit normal vector on $\partial\mathcal{B}$. We consider a rotationally symmetric two-dimensional synaptic weight kernel $w(\boldsymbol {x})=w(r)$ which satisfies $\int_{\mathbb{R}^{2}} \,\mathrm{d}\boldsymbol {x} w(\boldsymbol {x})= \mathcal{K}$, for some finite constant $\mathcal{K}$, and we introduce a function $g(\boldsymbol {x}):\mathbb{R}^{2} \rightarrow \mathbb{R}$ such that

52 $$ w(\boldsymbol {x})=(\nabla\cdot \boldsymbol {F}) (\boldsymbol {x})+g (\boldsymbol {x}). $$

Now considering a function $\varphi(r): \mathbb{R}^{+} \rightarrow \mathbb{R}$ which satisfies the condition $\lim_{r \rightarrow\infty} r \varphi (r)=0$, the vector field can be written using polar coordinates, that is, $\boldsymbol {F} = \varphi(r)(\cos\theta,\sin\theta) = \varphi(r) \boldsymbol {x}/ \vert \boldsymbol {x} \vert $ with $\boldsymbol {x}=r(\cos\theta,\sin\theta)$. Transforming the expressions $\mathcal{K}$ and *g* into polar coordinates, integrating Eq. (52), and using the divergence theorem, yields

53 $$\begin{aligned} \mathcal{K}& = \int_{0}^{\infty} \int_{0}^{2 \pi} r w(r, \theta) \,\mathrm{d}\theta\, \mathrm{d} r = \int_{0}^{\infty} \int_{0}^{2 \pi} r [ \nabla\cdot \boldsymbol {F} + g ](r, \theta) \,\mathrm{d}\theta\,\mathrm{d} r \end{aligned}$$

54 $$\begin{aligned} & = \oint \boldsymbol {F}\cdot \boldsymbol {n} \,\mathrm{d} s + \int_{0}^{\infty} \int_{0}^{2 \pi} r g(r,\theta) \,\mathrm{d}\theta\, \mathrm{d} r, \end{aligned}$$

where the line integral is described over a circle of radius $R\rightarrow\infty$. Therefore, the weight kernel can be written in the form

55 $$ \mathcal{K} = 2\pi\lim_{R \rightarrow\infty} R \varphi(R) + \int _{0}^{\infty} \int_{0}^{2 \pi} r g(r,\theta) \,\mathrm{d}\theta\, \mathrm{d} r. $$

Since the line integral vanishes, we may set $g(\boldsymbol {x})=\mathcal{K} \delta(\boldsymbol {x})$. We can now deduce the equation for $\varphi(r)$ by writing

56 $$\begin{aligned} w(r) ={}& \frac{\partial}{\partial r} \bigl[\varphi(r) \cos\theta \bigr] \frac{\partial r}{\partial x}+\frac{\partial}{\partial \theta} \bigl[\varphi(r) \cos\theta \bigr] \frac{\partial\theta }{\partial x} \\ &{} +\frac{\partial}{\partial r} \bigl[\varphi(r) \sin\theta \bigr]\frac{\partial r}{\partial y}+ \frac {\partial}{\partial\theta} \bigl[\varphi(r) \sin\theta \bigr] \frac{\partial\theta}{\partial y} \\ ={}&\frac{\partial \varphi}{\partial r}(r)+\frac{1}{r}\varphi(r),\quad r>0. \end{aligned}$$

The integration of (56) yields

57 $$ \varphi(r)=\frac{1}{r} \int_{\infty}^{r} x w(x) \,\mathrm{d} x. $$

Using the above results means that (23) can be evaluated as

58 $$\begin{aligned} \psi(\boldsymbol {x},t) &= \int_{\varOmega_{+}(t)} \,\mathrm{d}\boldsymbol {y} w \bigl( \vert \boldsymbol {x}-\boldsymbol {y} \vert \bigr) \\ &= \oint_{\partial\varOmega_{+}(t)} \,\mathrm{d} s \boldsymbol {F} \bigl( \bigl\vert \boldsymbol {x}- \boldsymbol { \gamma }(s) \bigr\vert \bigr) \cdot \boldsymbol {n}(s) + \mathcal{K} \int_{\varOmega_{+}(t)} \,\mathrm{d}\boldsymbol {y} \delta(\boldsymbol {x}-\boldsymbol {y}) \\ &= \oint_{\partial\varOmega_{+}(t)} \,\mathrm{d} s \varphi \bigl( \bigl\vert \boldsymbol {x}- \boldsymbol {\gamma}(s) \bigr\vert \bigr)\frac{\boldsymbol {x}-\boldsymbol{\gamma}(s)}{ \vert \boldsymbol {x}-\boldsymbol{\gamma}(s) \vert } \cdot \boldsymbol {n}(s) + \mathcal{K} C. \end{aligned}$$

Here $\boldsymbol {\gamma} \in\partial\varOmega_{+}$, and the integration over the Dirac-delta function gives $C = 1$ if ***x*** is within $\varOmega _{+}$, $C = 0$ if ***x*** is outside $\varOmega_{+}$, and $C = 1/2$ if ***x*** is on the boundary of $\varOmega_{+}$.

## Appendix 3: Numerical Scheme for the Interface Dynamics

Time stepping for interface dynamics requires a novel integration scheme. We present here the implementation used in our numerical experiments, which were found to be in agreement with the full spatio-temporal simulation, and we defer a numerical analytical study of its properties to a later date.

The method is formed of four constitutive parts: a scheme for approximating a closed curve (the interface $\partial\varOmega_{+}(t)$), a scheme to approximate the instantaneous normal velocity of the interface, a scheme to propagate the contour according to the normal velocity, and a strategy to remesh or postprocess the contour, if needed.

Closed contours: We chose a periodic parametrisation

$$\partial\varOmega_{+}(t) = \bigl\{ \boldsymbol {x} \in \mathbb{R}^{2} \colon x_{1}(t) = \xi _{1}(s,t), x_{2}(t) = \xi_{2}(s,t), s \in[0,2\pi) \bigr\} , $$

where $\xi_{1}$, $\xi_{2}$ are smooth and 2*π*-periodic in *s* for all *t*, and we approximated $\xi_{1}(s,\cdot)$, $\xi_{2}(s,\cdot)$ spectrally, using evenly spaced points in *s*. Using FFTs, we could approximate quickly and accurately the normal and tangent vectors to $\partial\varOmega_{+}$ at each point *s*, and each time *t*. We also parametrise the domain boundary *∂Ω* as follows:

$$\partial\varOmega= \bigl\{ \boldsymbol {x} \in \mathbb{R}^{2} \colon x_{1} = \eta_{1}(s), x_{2} = \eta_{2}(s), s \in[0,2\pi) \bigr\} , $$

where $\eta_{i}$ are continuous and 2*π*-periodic. We choose the paths *Γ* to be straight lines connecting ***x*** to its closest point on the boundary,

$$\varGamma(\boldsymbol {x}) = \Bigl\{ \boldsymbol {x}' \in \mathbb{R}^{2} \colon \boldsymbol {x}' = \boldsymbol {x} + s' \boldsymbol {\eta}_{*}, s' \in[0,1], \boldsymbol {\eta}_{*} = \arg\min_{s} \bigl\vert \boldsymbol {x} - \boldsymbol { \eta}(s) \bigr\vert \Bigr\} , $$

and we define ***ζ*** using the endpoints of *Γ*,

$$\boldsymbol {\zeta} \colon \boldsymbol {x} \mapsto \boldsymbol {x} + \boldsymbol {\eta}_{*}, \qquad \boldsymbol {\eta}_{*} = \arg\min _{s} \bigl\vert \boldsymbol {x} - \boldsymbol {\eta}(s) \bigr\vert . $$

Normal velocity: To simplify the discussion, let us consider the case where boundary conditions are not imposed. We compute the normal velocity by approximating the numerator and denominator of (21). The denominator is updated at each time step using (22) and (24): this requires the normal to $\partial\varOmega_{+}$ at time *t*, as well as the full history of $\partial\varOmega_{+}(t)$ in the interval $[0,t]$, in view of the integral (22). However, since $\eta(t) = \mathrm { e}^{-t} H(t)$, we found that retaining in memory and using only the last 20–50 time steps was not detrimental for the accuracy of the solution; we use the trapezium rule for both the integration along the contour in (24) (which is therefore spectrally accurate) and for the integral over $t'$ in (22). The numerator of (21) is computed using (26), for which a further integration along $\partial\varOmega_{+}$ is performed. The integrand is singular, and can be treated as in [48]. A similar strategy is used for (32) and (33), in the case of a bounded domain with Dirichlet boundary conditions. In all cases this step is by far the most time consuming of the algorithm, due to the large number of integrals which need to be evaluated at each step.

Position update: The contour is propagated in the normal direction, using the velocity computed at each point of the contour, $c_{n}(s,t)$. To this end we use a simple Euler update $\partial\varOmega_{+}(t+\Delta t) = \partial \varOmega_{+}(t) +c_{n} \Delta t$ to find the new contour, given that $c_{n}$ is also computed with $O(\Delta t)$ accuracy in time. Other choices are obviously possible, but require more function evaluations and more expensive quadrature rules. A stepsize of 0.05 or less has been used in our simulations.

Remeshing and postprocessing: The updated contour $\partial \varOmega_{+}(t+\Delta t)$ leads to a new parametrisation, that is, to an update of the functions $\xi_{1}(s,\cdot)$, $\xi_{2}(s,\cdot)$. Since we need a uniform distribution of the nodes with respect to the variable *s*, we redistribute points using standard interpolation [49]. As the pattern grows or shrinks, points are added or removed so as to keep the arclength between consecutive points approximately constant.

## Appendix 4: Geometric Formulae for a Piece-Wise Constant Kernel

Consider a portion of a disk whose upper boundary is a (circular) arc and whose lower boundary is a chord making a central angle $\phi_{0} < \pi$, illustrated as the shaded region in Fig. 6(A).

The area $A=A(r_{0},\phi_{0})$ of the (shaded) segment is then simply given by the area of the circular sector (the entire wedge-shaped portion) minus the area of an isosceles triangle, namely

59 $$ A(r_{0},\phi_{0}) = \frac{\phi_{0}}{2 \pi} \pi r_{0}^{2} - \frac{1}{2} r_{0} \sin( \phi_{0}/2) r_{0} \cos(\phi_{0}/2) = \frac{1}{2} r_{0}^{2} ( \phi _{0} - \sin \phi_{0} ). $$

The area of the overlap of two circles, as illustrated in Fig. 6(B), can be constructed as the total area of $A(r_{0},\phi_{0})+A(r_{1},\phi_{1})$. To determine the angles $\phi_{0,1}$ in terms of the centres, $(x_{0},y_{0})$ and $(x_{1},y_{1})$, and radii, $r_{0}$ and $r_{1}$, of the two circles we use the cosine formula, which relates the lengths of the three sides of a triangle formed by joining the centres of the circles to a point of intersection. Denoting the distance between the two centres by *d* where $d^{2} = (x_{0}-x_{1})^{2}+(y_{0}-y_{1})^{2}$,

60 $$ r_{1}^{2} = r_{0}^{2} + d^{2} - 2 r_{0} d \cos(\phi_{0}/2),\qquad r_{0}^{2} = r_{1}^{2} + d^{2} - 2 r_{1} d \cos(\phi_{1}/2). $$

Hence

61 $$\begin{aligned} \begin{aligned} \phi_{0}(d,r_{1})={}& 2 \cos^{-1} \biggl( \frac{r_{0}^{2} + d^{2} - r_{1}^{2}}{2 r_{0} d} \biggr), \\ \phi_{1}(d,r_{1})={}& 2 \cos^{-1} \biggl( \frac{r_{1}^{2} + d^{2} - r_{0}^{2}}{2 r_{1} d} \biggr). \end{aligned} \end{aligned}$$
